# Microbiomics and metabolomics reveal microbial–metabolic signatures associated with body weight variation in chickens

**DOI:** 10.1016/j.psj.2026.107117

**Published:** 2026-05-13

**Authors:** Xudong Zhao, Yingping Tian, Sheng Wu, Yong Yue, Yun Du, Fuping Zhang, Xing Lei, Zhiwen Chen, Yaozhou Jiang, Qinsong Liu

**Affiliations:** a Key Laboratory of Animal Genetics, Breeding and Reproduction in the Plateau Mountainous Region, Ministry of Education, College of Animal Science, Guizhou University, Guiyang 550025 Guizhou Province, China; b School of Animal Technology and Innovation, Institute of Agricultural Technology, Suranaree University of Technology, Nakhon Ratchasima 30000, Thailand

**Keywords:** Chicken, Body weight, Gut microbiota, Serum metabolomics, Multi-omics

## Abstract

Body weight is a complex quantitative trait regulated by the combined effects of polygenic inheritance and environmental factors. Among non-genetic determinants, the gut microbiota and its associated metabolic processes play critical roles in influencing growth performance in poultry. However, the microbiota–metabolite associations underlying growth-related phenotypes in chickens with divergent body weights remain insufficiently characterized. In this study, we integrated 16S rRNA gene sequencing with untargeted serum metabolomics to systematically characterize the cecal microbial communities and host metabolic profiles associated with body weight variation in hybrid chickens. Our results showed that significant divergence in body weight between groups emerged from 8 weeks of age onward. The high-body-weight group was significantly enriched in potentially functional taxa, including *Lactobacillus, Subdoligranulum, Megasphaera, Negativibacillus,* and *Sutterella*. Metabolomic analysis identified 60 differentially abundant metabolites. The high-body-weight group was characterized by upregulation of glycerophospholipid metabolites and enrichment of lipid metabolism pathways, whereas the low-body-weight group exhibited enrichment primarily in amino acid and pyrimidine metabolism pathways. Correlation analysis revealed that taxa enriched in the high-body-weight group were positively associated with lipid-related metabolites, whereas characteristic taxa of the low-body-weight group, such as *Colidextribacter* and *Pseudoflavonifractor*, were positively correlated with specific amino acid-related metabolites. Collectively, these findings provide novel multi-omics evidence of microbiota–metabolite associations underlying growth traits in poultry and offer potential insights into the development of microbiota-based strategies to improve production performance.

## Introduction

Poultry represents an accessible and high-quality source of animal protein across diverse production systems and cultural contexts. Its production and consumption continue to expand globally, underscoring its critical role in ensuring nutritional security and advancing sustainable agricultural development (Mottet et al., 2017; Nassar, 2026). In this context, the increasing global demand for poultry meat has intensified the need for efficient and cost-effective strategies to improve broiler growth performance. The Guizhou Yellow chicken, a representative indigenous dual-purpose breed, is characterized by strong environmental adaptability and superior meat quality; however, its growth performance remains markedly inferior to that of commercial broiler lines, such as the Anka chicken (Wu et al., 2024; Tian et al., 2025). Although crossbreeding with commercial strains can partially improve body weight traits, substantial inter-individual variation persists within hybrid populations, and the underlying regulatory mechanisms remain poorly understood.

Body weight is a complex quantitative trait shaped by the interplay between polygenic genetic effects and environmental factors (Zhong et al., 2024; Niu et al., 2025). Despite substantial advances in genomics that have improved understanding of the genetic basis of growth traits, a considerable proportion of phenotypic variance cannot be fully explained by host genetics alone (Guan et al., 2025; Zhu et al., 2025). This gap highlights the importance of environmental and non-genetic factors in regulating growth. Among these factors, the gut microbiota has emerged as a critical modulator. Its compositional and functional variability is closely associated with host growth phenotypes and can directly influence growth outcomes, as demonstrated by interventions such as fecal microbiota transplantation (Ma et al., 2023). Growing evidence indicates that the gut microbiota regulates host growth through multiple interconnected mechanisms, including modulation of nutrient metabolism (Aruwa et al., 2021; Wen et al., 2021), production of bioactive metabolites (Khan et al., 2020; Cox et al., 2022), and regulation of immune homeostasis (Round and Mazmanian, 2009; Oke et al., 2025). For example, the relative abundances of *Microbacterium* and *Sphingomonas* are elevated in the ceca of high-body-weight chickens, whereas *Slackia* is enriched in low-body-weight individuals (Zhang et al., 2022). In addition, an increased abundance of *Lactobacillus* and *Bifidobacterium* in the jejunum has been positively associated with enhanced body weight, potentially through the mitigation of intestinal inflammation (Zhang et al., 2022). Consistently, dietary supplementation with probiotics—including *Bacillus subtilis, Lactobacillus plantarum*, and *Paenibacillus polymyxa*—has been shown to significantly improve growth performance and feed efficiency in broilers (Bai et al., 2017; Wang et al., 2021).

Notably, the regulatory influence of the gut microbiota on host metabolism extends beyond the intestinal environment. Microbially derived or microbiota-regulated metabolites can enter the systemic circulation and exert distal, organism-wide effects (Visconti et al., 2019). Recent multi-omics studies have demonstrated robust associations between gut microbial composition and host serum metabolomic profiles, indicating that the abundance of numerous circulating metabolites is directly or indirectly shaped by the gut microbiota. Perturbations in microbial community structure can consequently drive extensive remodeling of the serum metabolome, thereby contributing to systemic metabolic regulation via circulating metabolites (Krautkramer et al., 2021; Murga-Garrido et al., 2021). Evidence from indigenous chicken breeds further supports this integrative framework. In Guizhou Yellow chickens, microbial taxa enriched in high-body-weight individuals show significant positive correlations with metabolites such as pantothenic acid (vitamin B5) and menadione (vitamin K3) (Yang et al., 2023). Similarly, studies in Qiandongnan Xiaoxiang chickens have shown that *Sphaerochaeta* may enhance growth performance by promoting bovinic acid metabolism, whereas *Synergistes* and members of the *Desulfovibrionaceae* family may suppress growth through the induction of pro-inflammatory cytokines (Wang et al., 2023). Collectively, these findings position the serum metabolome as a critical intermediary linking the gut microbiota to host phenotypes, providing a systems-level perspective on metabolic status and its regulatory mechanisms. Accordingly, integrative analyses combining microbiome and serum metabolome data provide a powerful framework for elucidating microbiota-mediated metabolic networks and advancing our understanding of the molecular mechanisms underlying body weight variation.

In the present study, we employed hybrid progeny derived from the Anka males and the Guizhou Yellow females as the experimental model. The phenotypic divergence observed within this population—arising under a shared genetic background and uniform environmental conditions—provides a robust system for dissecting the contributions of non-genetic regulatory factors, including the gut microbiota and host metabolic processes, to complex traits. By integrating high-throughput 16S rRNA gene sequencing with untargeted serum metabolomics, we aimed to identify key microbial taxa and metabolic signatures associated with body weight variation and to systematically characterize the relationships among growth phenotypes, cecal microbial communities, and circulating metabolite profiles. This study not only provides new insights into the biological basis of growth and development in broilers, but also offers a theoretical framework for improving poultry production performance through targeted manipulation of the gut microbiota.

## Materials and methods

### Experimental animals and management

A total of 120 one-day-old hybrid chicks (Anka ♂ × Guizhou Yellow ♀), with equal numbers of males and females, were used in this study. All birds were reared under identical environmental and nutritional conditions at the Experimental Poultry Farm of Guizhou University for 18 weeks. At the beginning of the experiment, all birds were individually identified using wing bands and randomly allocated to cages. Stocking density was adjusted according to developmental stage to ensure adequate space allowance as birds grew. No birds were intentionally removed or selected prior to the final body weight-based grouping at 18 weeks of age. From 0 to 4 weeks of age, 16 randomly assigned mixed-sex birds were housed per cage; from 4 to 10 weeks, the same birds were randomly redistributed into cages with 8 birds per cage; and from 10 to 18 weeks, all birds were housed individually in three-tier stepped cages. Environmental conditions were controlled as follows: relative humidity was maintained at 60–65% during the first 7 days and subsequently reduced to 55–60%. Ambient temperature was maintained at 33–35°C during the first 3 days of brooding, decreased to 32–34°C from days 4 to 7, and then gradually reduced by 2–3°C per week until reaching approximately room temperature (∼20°C), which was maintained thereafter. Lighting was managed according to a staged program: chicks were exposed to 24 h of light at 20 lx during the first 2 days; from days 3 to 7, the photoperiod was reduced by 1 h per day until reaching 18 h of light at 20 lx; from day 8 to day 119, a constant 8 h light regimen was maintained at 10 lx; and from day 120 onward, the photoperiod was increased to 10 h per day at 10 lx. Throughout the experiment, birds had ad libitum access to feed and water, and routine immunizations were administered according to standard husbandry practices. The basal diets were formulated according to the Nutrient Requirements of Yellow-Feathered Broilers (NY/T 3645–2020) (Table 1). Different diets were provided according to growth stage, including starter (0–3 weeks), grower (4–6 weeks), and finisher (7–18 weeks) diets.

**Table 1:** Basal diet composition and nutrient level (air-dry basis).

Items Ingredients %	Content		
	0-3 weeks	4-6 weeks	7-18 weeks
Corn	64.00	70.00	58.00
Soybean meal	28.00	21.00	16.00
Corn germ	4.00	5.00	10.50
Wheat bran	0.00	0.00	11.00
Limestone	1.05	1.10	1.70
CaHPO_4_	0.71	0.63	0.97
NaCl	0.22	0.15	0.20
montmorillonite	0.20	0.30	0.20
Lysine	0.60	0.60	0.21
Methionine	0.10	0.10	0.10
Threonine	0.12	0.12	0.12
Premix^1^	1.00	1.00	1.00
Total	100	100	100
Nutritional level %			
Metabolizable energy^2^ (MJ/kg)	12.17	12.26	11.51
Crude protein	19.34	16.71	15.79
Calcium	0.84	0.68	0.97
Available phosphorus	0.30	0.23	0.32
Lysine	1.34	1.06	0.86

1 The premix provided per kilogram of feed included: vitamin A, 4,000 IU; vitamin D₃, 3,600 IU; vitamin E, 15.5 IU; vitamin K, 2.1 mg; vitamin B₁ (thiamine), 2.6 mg; vitamin B₂ (riboflavin), 5.0 mg; vitamin B₆ (pyridoxine), 3.0 mg; vitamin B₁₂ (cyanocobalamin), 0.01 mg; folic acid, 0.45 mg; pantothenic acid, 12.2 mg; choline chloride, 500 mg; calcium formate, 65 mg; a compound enzyme, 100 mg; and sodium bicarbonate (NaHCO₃), 1,000 mg.

2 Metabolic energy was a calculated value, while the others were measured values.

### Body weight measurement and grouping

During the experimental period, body weight of all birds was measured at two-week intervals following an 8 h fasting period. Birds were also fasted for 8 h prior to sample collection at 18 weeks of age. No grouping was performed before the end of the experiment. At 18 weeks of age, birds were retrospectively ranked according to body weight within each sex, and individuals with extreme phenotypes were selected for subsequent analyses. Specifically, the six heaviest males and six heaviest females (n = 12) were assigned to the high body weight group (H group), whereas the six lightest males and six lightest females (n = 12) were assigned to the low body weight group (L group). These selected individuals were subsequently used for serum metabolomics and cecal microbiota analyses.

### Sample collection

Blood samples (10 mL) were collected from all selected birds in the H and L groups (n = 24) via the wing vein. Samples were centrifuged at 3,000 × g for 10 min at 4°C (Dunn et al., 2011), after which the serum was separated, aliquoted, and stored at −80°C until further analysis. Following blood collection, birds were humanely euthanized by electrical stunning followed by exsanguination via cervical bleeding, in accordance with established animal welfare guidelines. Under sterile conditions, the ceca were carefully excised, and cecal contents were collected. The collected samples were immediately snap-frozen in liquid nitrogen and stored at −80°C for subsequent analyses.

### 16S rRNA gene sequencing of cecal microbiota

Total genomic DNA was extracted from cecal content samples using the PowerSoil DNA Isolation Kit (MO BIO Laboratories, USA) according to the manufacturer’s instructions. DNA concentration and purity were assessed using a NanoDrop 2000 UV–Vis spectrophotometer (Thermo Fisher Scientific, Wilmington, USA). The V3–V4 hypervariable regions of the bacterial 16S rRNA gene were amplified using the primer pair 338F (5′-ACTCCTACGGGAGGCAGCA-3′) and 806R (5′-GGACTACHVGGGTWTCTAAT-3′). PCR amplification was performed using a two-step protocol: the first round involved specific amplification of the V3–V4 region, and the second round incorporated sequencing adapters and barcode sequences. Detailed reaction mixtures and thermal cycling conditions are provided in Supplementary Tables S1–S4. Following amplification, PCR products were purified and quantified using the Quant-iT™ dsDNA High-Sensitivity Assay Kit. Equimolar amounts of purified amplicons from each sample were pooled and subjected to paired-end sequencing (2 × 250 bp) on an Illumina HiSeq 2500 platform.

### Bioinformatic analysis

Raw paired-end sequencing reads were quality-filtered and trimmed using the DADA2 plugin implemented in QIIME2 (Version 2020.6), which performs sequence denoising, error correction, paired-end merging, and chimera removal to generate high-quality amplicon sequence variants (ASVs). ASVs with extremely low abundance (<0.005% of total sequences) were removed to reduce potential noise. Representative ASV sequences were taxonomically assigned against the SILVA reference database (release 138) using the naïve Bayes classifier (classify-sklearn) implemented in QIIME2 with a confidence threshold of 70%. Alpha diversity indices were calculated from the ASV abundance table to assess within-sample microbial diversity, whereas beta diversity was evaluated using principal coordinates analysis (PCoA) based on distance matrices to compare microbial community structures among samples. Differentially abundant taxa among groups were identified using linear discriminant analysis effect size (LEfSe).

### Metabolomic analysis

***Metabolite Extraction.*** Serum samples (100 μL) were mixed with 400 μL of extraction solvent (methanol:acetonitrile, 1:1, v/v) containing an isotopically labeled internal standard mixture. Samples were vortexed for 30 s, followed by ultrasonication in an ice–water bath for 10 min, and then incubated at −40°C for 1 h to facilitate protein precipitation. Subsequently, samples were centrifuged at 13,800 × g for 15 min at 4°C, and the supernatants were collected for analysis. Equal aliquots of supernatant from each sample were pooled to generate quality control (QC) samples, which were analyzed alongside the experimental samples to monitor the stability and reproducibility of the analytical process.

***UPLC–MS/MS Analysis.*** Chromatographic separation was performed using a Vanquish ultra-high-performance liquid chromatography (UHPLC) system (Thermo Fisher Scientific) equipped with a Waters ACQUITY UPLC BEH Amide column (2.1 mm × 50 mm, 1.7 μm). The mobile phase consisted of (A) an aqueous solution containing 25 mmol/L ammonium acetate and 25 mmol/L ammonia and (B) acetonitrile. The autosampler temperature was maintained at 4°C, and the injection volume was set to 2 μL. Mass spectrometric analysis was performed using an Orbitrap Exploris 120 mass spectrometer (Thermo Fisher Scientific). Both MS1 and MS/MS data were acquired using Xcalibur software (Version 4.4). The key operating parameters were as follows: sheath gas flow rate, 50 arbitrary units (Arb); auxiliary gas flow rate, 15 Arb; capillary temperature, 320°C; MS1 resolution, 60,000; MS/MS resolution, 15,000; and stepped normalized collision energy (NCE), 20/30/40. The spray voltage was set to 3.8 kV in positive ion mode and −3.4 kV in negative ion mode.

***Data Preprocessing and Annotation.*** Raw mass spectrometry data were converted to mzXML format using ProteoWizard. Data preprocessing—including peak detection, extraction, alignment, and integration—was performed using a custom R package based on the XCMS platform. Metabolite annotation was performed by matching MS/MS spectra against the BiotreeDB database (Version 2.1), with a similarity score threshold of 0.3 for metabolite identification.

***Data Analysis.*** Principal component analysis (PCA) was used to assess sample distribution and clustering. Differential metabolites between groups were identified based on fold change (FC) and statistical testing. Orthogonal partial least squares discriminant analysis (OPLS-DA) was performed using SIMCA software (Version 16.0.2) after data normalization and scaling. Model robustness was evaluated by 200 permutation tests and cross-validation. Variable importance in projection (VIP) scores were calculated to assess metabolite contributions. Metabolites with FC > 1, *P* < 0.05, and VIP > 1 were defined as differential metabolites. KEGG pathway enrichment analysis was performed using a hypergeometric test based on the KEGG database.

### Statistical analysis

Statistical analyses were performed using IBM SPSS Statistics (Version 27.0; IBM Corp., Armonk, NY, USA). Differences in body weight between groups were evaluated using independent-samples *t*-tests. Results are presented as the mean ± standard error (SE), and a *P* value < 0.05 was considered statistically significant. Spearman’s rank correlation analysis between microbial taxa and serum metabolites was performed in R (Version 4.4.1) using the *stats* package. The correlation matrix was visualized as a heatmap using the *pheatmap* package.

## Results

### Growth performance of the chickens

**Fig. 1:**
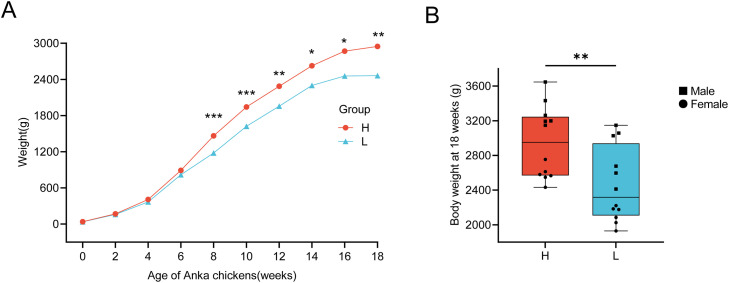
Body weights of hybrid chickens. (**A**) Changes in body weight of ages 0 to 18 weeks. * *P* < 0.05, ** *P* < 0.01, *** *P* < 0.001. (**B**) Body weight distribution of experimental chickens at 18 weeks of age. H, high weight chicken group (n = 12). L, low weight chicken group (n = 12).

The trajectories of body weight gain from 0 to 18 weeks were broadly similar between the H and L groups. No differences in body weight was observed during the first 6 weeks under identical rearing conditions. In contrast, body weight diverged between the two groups from 8 weeks onward (*P* < 0.01). At 18 weeks, chickens in the H group exhibited greater body weight than those in the L group (2947.70 ± 118.08 g vs. 2461.48 ± 124.48 g, *P* < 0.01) (Fig. 1) (Table 2).

**Table 2:** Comparison of body weight differences between the H and L groups at different weeks of age.

Age (week)	H (g) Mean ± SE	L (g) Mean ± SE	t-value	*P*-value
0	38.22 ± 1.19	37.37±1.53	0.439	0.665
2	169.38 ± 6.18	160.12 ± 8.96	0.851	0.404
4	407.25 ± 12.75	367.33 ± 27.07	1.334	0.196
6	890.50 ± 21.54	817.25 ± 46.22	1.437	0.165
8	1463.90 ± 39.29^A^	1178.77 ± 51.27^B^	4.414	0.000
10	1942.79 ± 56.28^A^	1622.30 ± 62.95^B^	3.796	0.001
12	2286.89 ± 75.35^A^	1956.90 ± 79.14^B^	3.020	0.006
14	2625.90 ± 100.25^a^	2300.54 ± 106.55^b^	2.224	0.037
16	2870.02 ± 107.83^a^	2456.92 ± 125.06^b^	2.502	0.020
18	2947.70 ± 118.08^A^	2461.48 ± 124.48^B^	2.834	0.010

Note: H: High weight group; L: Low weight group.

Means within a row with different lowercase letters (a, b) differ significantly (*P* < 0.05), and those with different uppercase letters (A, B) differ highly significantly (*P* < 0.01).

### Cecal microbial diversity between the H and L groups

**Fig. 2:**
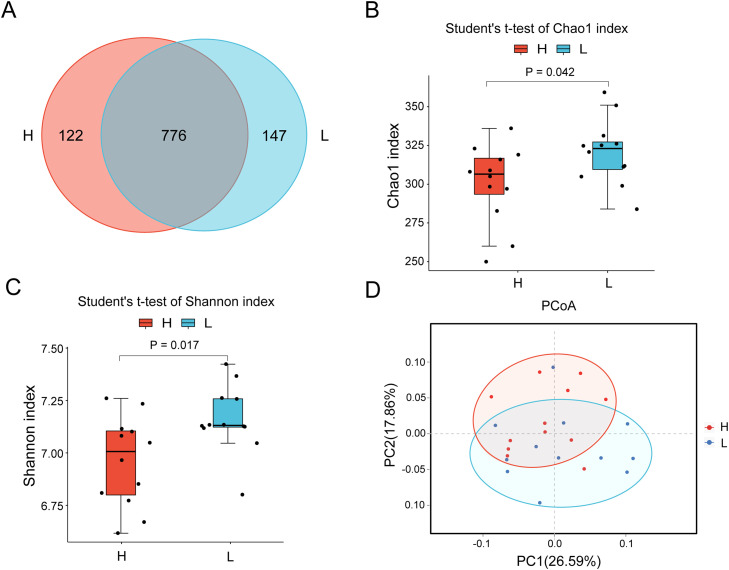
Diversity analysis of the gut microbiota. (A) Venn diagram showing shared and unique amplicon sequence variants (ASVs) between the H and L groups. (B, C) Alpha diversity of the intestinal microbiota, as assessed by the Chao1 and Shannon indices. Boxplots represent the median, interquartile range (IQR), and range (min–max). Statistical significance between groups was evaluated using Student’s t-test (* *P* < 0.05, ** *P* < 0.01). (D) Principal coordinate analysis (PCoA) based on weighted UniFrac distances showing the beta diversity of microbial communities. Each point represents an individual sample, and ellipses indicate the 95% confidence interval for each group.

To characterize differences in the gut microbiota between chickens with divergent body weights, 16S rRNA gene sequencing was performed on cecal content samples from the H and L groups. A total of 1,940,734 raw paired-end reads were generated, of which 1,787,247 high-quality clean reads were retained after quality filtering and merging. Sequencing depth was high across all samples, with a minimum of 73,232 reads and an average of 74,469 reads per sample (Table S5). At the amplicon sequence variant (ASV) level, a total of 1,045 ASVs were identified across the two groups, including 776 shared ASVs, 147 unique ASVs in the L group, and 122 unique ASVs in the H group (Fig. 2A). Rarefaction curves approached saturation when the sequencing depth reached 20,000 reads, indicating that the sequencing effort was sufficient to capture the majority of microbial diversity in each sample, with only a limited number of low-abundance ASVs remaining undetected (Fig. S1). Alpha diversity analysis revealed that both the Shannon and Chao1 indices were higher in the L group than in the H group (*P* = 0.017 and *P* = 0.042, respectively), indicating greater microbial richness and diversity in the L group (Fig. 2B, C). Principal coordinates analysis (PCoA) based on weighted UniFrac distances further revealed a partial separation of microbial community structure between the two groups, with PC1 and PC2 explaining 26.59% and 17.86% of the total variance, respectively (Fig. 2D).

### Comparison of gut microbiota between the H and L groups

**Fig. 3:**
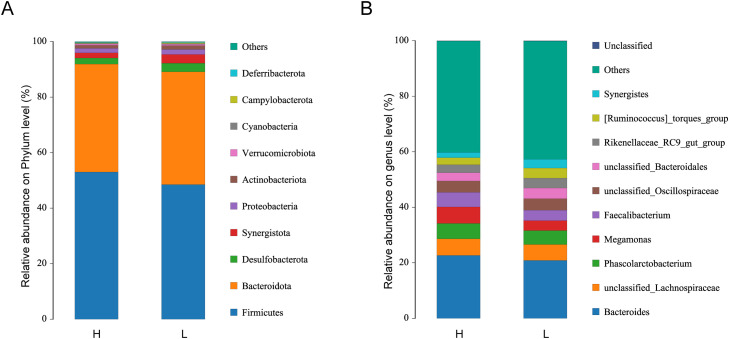
Cecal microbial species composition. (A) Relative abundance of gut microbiota at phylum level. (B) Relative abundance of gut microbiota at genus level.

To further compare gut microbial composition between the H and L groups, taxonomic profiles were analyzed at both the phylum and genus levels. At the phylum level, Firmicutes and Bacteroidetes were the dominant phyla in both groups, together accounting for more than 80% of the total relative abundance. Additional phyla with relative abundances exceeding 1% included *Desulfobacterota, Synergistota, Proteobacteria*, and *Actinobacteriota* (Fig. 3A).

At the genus level, the top 10 most abundant taxa in both groups were Bacteroides, unclassified Lachnospiraceae, Megamonas, Phascolarctobacterium, Faecalibacterium, unclassified Oscillospiraceae, unclassified Bacteroidales, Rikenellaceae_RC9_gut_group, [Ruminococcus]_torques_group, and Synergistes. Comparative analysis revealed notable differences between groups: the relative abundances of Megamonas and Faecalibacterium were higher in the H group than in the L group, with increases of 2.25% and 1.60%, respectively. In contrast, Synergistes was more abundant in the L group, exhibiting a 1.25% higher relative abundance than in the H group (Fig. 3B).

### Differential microbial taxa between the H and L groups

**Fig. 4:**
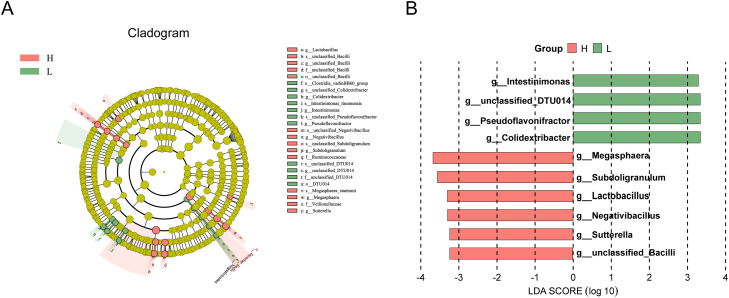
(A) Evolutionary branching diagram for LEfSe analysis. (B) Histogram of the distribution of LDA values.

LEfSe analysis was performed to identify microbial taxa that were differentially enriched between the H and L groups (LDA score > 2). A total of 15 discriminative taxa were enriched in the H group, whereas 12 taxa were enriched in the L group. At the genus level, six taxa—unclassified *Bacilli, Sutterella, Negativibacillus, Lactobacillus, Subdoligranulum*, and *Megasphaera*—were significantly more abundant in the H group than in the L group. In contrast, *Colidextribacter, Pseudoflavonifractor*, unclassified *DTU014*, and *Intestinimonas* were significantly enriched in the L group (Fig. 4). Notably, some of the discriminative taxa identified in both the H and L groups were annotated at higher taxonomic levels (e.g., phylum, family, or order). Therefore, to ensure biological interpretability and avoid overgeneralization, subsequent analyses and discussion in this study primarily focused on taxa identified at the genus level.

### Serum differential metabolites between the H and L groups

**Fig. 5:**
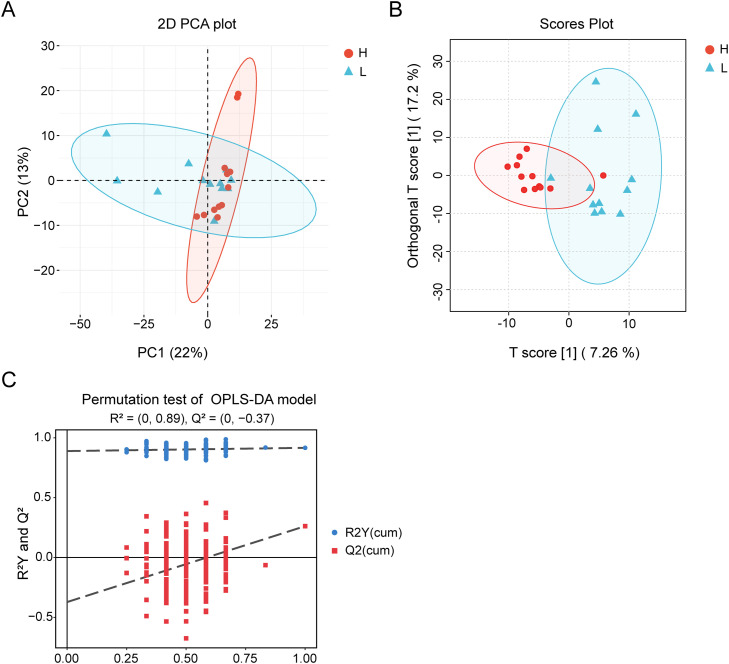
Metabolite profiling of serum by untargeted UPLC-MS/MS metabolomics. (A) PCA analyses. (B) Orthogonal partial least squares discriminant analysis (OPLS-DA). (C) OPLS-DA model replacement test plot.

Untargeted metabolomic analysis of 24 serum samples from the H and L groups identified a total of 539 annotated metabolites, including 359 detected in positive ion mode and 180 in negative ion mode. Principal component analysis (PCA) revealed a trend toward separation between the H and L groups, indicating distinct global metabolic profiles (Fig. 5A). To further characterize these differences, an OPLS-DA model was constructed. The resulting score plot demonstrated a clear separation between groups, highlighting pronounced metabolic divergence associated with body weight (Fig. 5B). Model validation using permutation testing yielded an R² intercept of 0.89 and a Q² intercept of −0.37. Importantly, the Q² regression line below zero, confirming the absence of overfitting and indicating strong model stability and predictive reliability (Fig. 5C).

Metabolites identified in both positive and negative ion modes were combined for subsequent analyses. Differential metabolites between the H and L groups were screened based on the criteria of VIP > 1 and *P* < 0.05. In total, 60 significantly differential metabolites were identified between the two groups (Table S6). Compared with the L group, metabolites belonging to the glycerophospholipid class (e.g., PE (22:4(7Z,10Z,13Z,16Z)/16:1(9Z)) and PC (20:3(8Z,11Z,14Z)/20:1(11Z))) and benzene and substituted derivatives (e.g., 2,6-dihydroxybenzoic acid and gallic acid) were generally upregulated in the H group. In contrast, metabolites classified as carboxylic acids and derivatives—including citrulline, argininic acid, and D-proline—were more abundant in the L group (Table 3).

**Table 3:** Top 20 differential serum metabolites between H and L groups.

Metabolite	FC (H/L)	log_2_ FC	*P-value*	VIP	Regulted
Citrulline	0.29	−1.80	0.008	2.62	down
PE (22:4(7Z,10Z,13Z,16Z)/16:1(9Z))	2.24	1.16	0.002	2.48	up
2,6-Dihydroxybenzoic acid	3.65	1.87	0.011	2.36	up
Gallic acid	2.30	1.20	0.020	2.32	up
Argininic acid	0.37	−1.44	0.015	2.31	down
PC (20:3(8Z,11Z,14Z)/20:1(11Z))	1.37	0.46	0.008	2.27	up
PC (18:2(9Z,12Z)/20:5(5Z,8Z,11Z,14Z,17Z))	1.17	0.77	0.006	2.23	up
D-Proline	0.81	−0.31	0.013	2.22	down
LY294002	2.07	1.05	0.001	2.21	up
Creatine	0.43	−1.21	0.016	2.20	down
N-Nitroso-pyrrolidine	1.39	0.48	0.003	2.19	up
Propionic acid	0.73	−0.46	0.013	2.18	down
Galanthamine N-Oxide	1.56	0.64	0.003	2.16	up
Raddeanin A	0.83	−0.28	0.024	2.11	down
PC (20:1(11Z)/14:1(9Z))	1.53	0.61	0.021	2.06	up
L-allo-isoleucine	0.80	−0.32	0.009	2.03	down
L-Allothreonine	0.61	−0.70	0.011	2.03	down
Arachidonic Acid (peroxide free)	1.56	0.64	0.004	2.02	up
Methoxypyrazine	1.31	0.39	0.020	2.02	up
Glycolic acid	0.88	−0.19	0.009	1.98	up

Note: H: High weight group; L: Low weight group.

VIP values were calculated based on the OPLS-DA model.

Differential metabolites were identified with the criteria: VIP > 1 and *P* < 0.05.

### KEGG pathway enrichment analysis

**Fig. 6:**
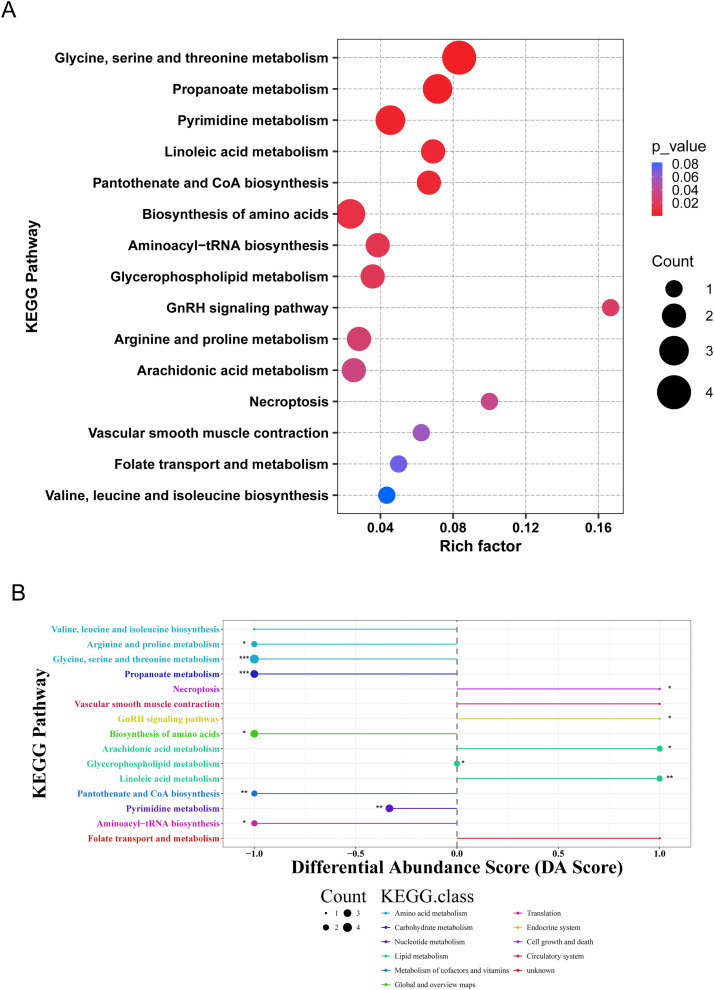
KEGG pathway enrichment analysis. (A) Differential metabolite KEGG enrichment plot. (B) Differential abundance score plot.

To explore the metabolic pathways associated with the identified differential metabolites, KEGG enrichment analysis was performed. The results indicated that these metabolites were primarily enriched in pathways related to amino acid, lipid, and nucleotide metabolism. Further analysis revealed distinct pathway enrichment patterns between the two groups. Lipid metabolism–related pathways, including glycerophospholipid metabolism and linoleic acid metabolism, were enriched in the H group. In contrast, pathways associated with amino acid metabolism, nucleotide metabolism, and pantothenate and coenzyme A biosynthesis—such as glycine, serine, and threonine metabolism, pyrimidine metabolism, and pantothenate and CoA biosynthesis—were enriched in the L group. These findings indicate differences in metabolic pathway enrichment between chickens with divergent body weights (Fig. 6).

### Spearman correlation analysis

**Fig. 7:**
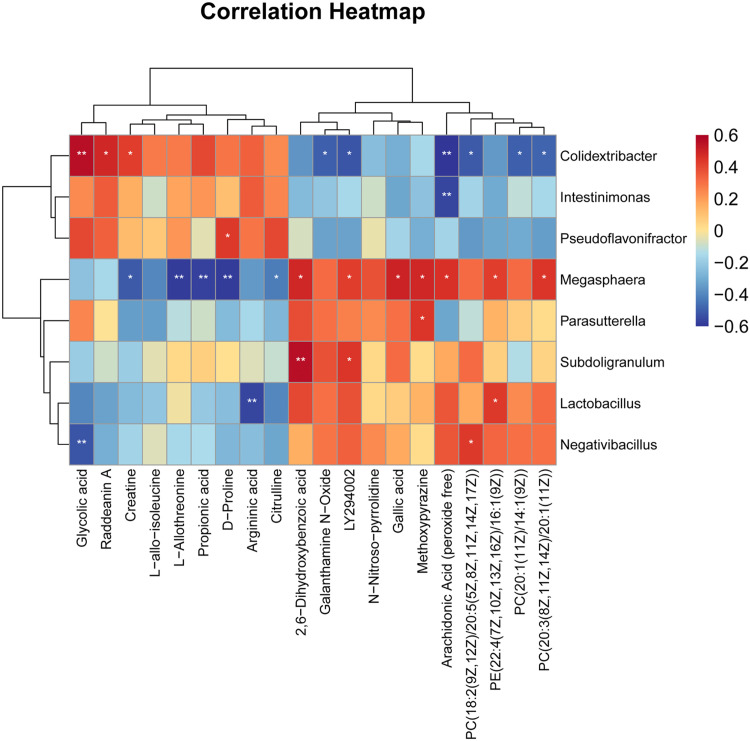
Differential metabolite-differential microbe correlation heat map. Note: Differential metabolites are on the x-axis. Differential microorganisms are on the y-axis. Red colour indicates a positive correlation, and blue colour indicates a negative correlation.

To investigate associations between gut microbiota and serum metabolites, a correlation network was constructed based on Spearman’s rank correlation analysis between microbial genera and differential metabolites (Fig. 7). The results revealed significant positive and negative correlations between specific bacterial taxa and metabolites, with clear clustering patterns. Specifically, *Megasphaera* was positively correlated with arachidonic acid (peroxide free) (*P* < 0.05). In addition, *Megasphaera* and *Lactobacillus* were positively correlated with PE (22:4(7Z,10Z,13Z,16Z)/16:1(9Z)) (*P* < 0.05), whereas *Megasphaera* and *Negativibacillus* were positively associated with PC (18:2(9Z,12Z)/20:5(5Z,8Z,11Z,14Z,17Z)) (*P* < 0.05). Moreover, *Colidextribacter* was positively correlated with glycolic acid, raddeanin A, and citrulline (*P* < 0.05), but negatively correlated with PC (20:1(11Z)/14:1(9Z)) and arachidonic acid (peroxide free) (*P* < 0.05). Overall, these results indicate significant associations between specific gut microbial taxa and host serum metabolic profiles.

## Discussion

Body weight is a key trait in poultry production, as it reflects growth performance and directly influences production efficiency and economic return (Mebratie et al., 2019; Monteiro et al., 2026). In this study, we systematically investigated the relationships among body weight variation, cecal microbial composition, and serum metabolomic profiles in hybrid chickens. Our results indicate that divergence in body weight is accompanied by marked remodeling of the gut microbiota and coordinated metabolic reprogramming in the host. High-body-weight chickens were characterized by the enrichment of functionally advantageous microbial taxa and enhanced lipid metabolism, whereas low-body-weight chickens exhibited greater microbial diversity and a metabolic profile more strongly associated with amino acid metabolism. Collectively, these findings support the notion that the gut microbiota contributes to body weight variation, at least in part, by modulating key host metabolic pathways.

Growth in poultry is a complex process governed by interactions between genetic potential and nutritional supply. During early development, genetic background (Manjula et al., 2018; Dou et al., 2022) and basal nutritional conditions (Gaweł et al., 2022; Zhao et al., 2023) play dominant roles in shaping body weight. As development proceeds, growth outcomes become increasingly dependent on the regulation of energy allocation and metabolic networks, such that genetic and nutritional factors alone are insufficient to fully explain phenotypic divergence. Consistent with this framework, no significant differences in body weight were observed between the H and L groups during the first 6 weeks, whereas a clear divergence emerged from 8 weeks onward and continued to widen thereafter. This pattern suggests that body weight differentiation primarily occurred during the mid-to-late growth stages. Previous studies have shown that the broiler gut microbiota undergoes dynamic succession throughout development, progressing from rapid early colonization to intermediate restructuring and eventual stabilization (Cheng et al., 2025). Because this maturation process is closely linked to host metabolic regulatory capacity (Liu et al., 2021; Bajagai et al., 2024), the body weight divergence observed in later stages may be partly attributable to age-dependent shifts in the gut microbiota and its increasingly prominent role in host metabolic regulation.

Notably, the L group exhibited significantly higher cecal microbial richness and diversity than the H group, indicating that greater microbial diversity does not necessarily correspond to improved growth performance. Previous studies have demonstrated substantial variability in the relationship between gut microbiota and production performance in poultry, with microbial features associated with high productivity differing across studies (Marková et al., 2024). Rather than simply exhibiting higher community diversity, the gut microbiota of high-performance individuals may be more function-oriented, whereby the enrichment of dominant functional taxa enhances nutrient digestion, absorption, and energy utilization efficiency. In contrast, low-performance individuals may exhibit higher α-diversity or more complex community structures; however, these characteristics do not necessarily translate into improved production efficiency (Bae et al., 2017). Furthermore, some studies have reported that higher feed efficiency may be associated with reduced microbial richness or a more streamlined functional structure, potentially reflecting increased functional specialization and more efficient resource utilization within the microbial community (Stanley et al., 2012; Liu et al., 2021). Therefore, elevated microbial diversity may reflect a more complex ecological network and intensified niche competition, potentially leading to dispersed resource utilization and reduced overall metabolic efficiency. In contrast, high-body-weight chickens may achieve more efficient metabolic regulation through the enrichment of key functional taxa, thereby promoting enhanced growth performance.

At the taxonomic level, several genera enriched in the H group, including *Lactobacillus, Subdoligranulum, Megasphaera*, and *Sutterella*, are closely associated with the production and metabolism of short-chain fatty acids (SCFAs) (Deleu et al., 2021; Mukhopadhya and Louis, 2025). As major end-products of microbial fermentation, SCFAs serve as important energy substrates for the host, particularly for intestinal epithelial cells, and play essential roles in maintaining gut function and immune homeostasis. Previous studies have shown that SCFAs promote epithelial proliferation and villus development, thereby enhancing intestinal absorptive capacity, and improve nutrient assimilation by modulating the gut microenvironment and digestive processes (Koh et al., 2016; Morrison and Preston, 2016; Liu et al., 2021). In addition, SCFAs promote the differentiation of regulatory T cells and suppress inflammatory responses (Smith et al., 2013; Kim, 2021), thereby reducing immune-associated energy expenditure. Of particular interest, *Lactobacillus* and *Megasphaera* were co-enriched in the H group, suggesting a potential cooperative interaction. *Lactobacillus* can ferment carbohydrates to produce lactate (Ağagündüz et al., 2021), which may then be utilized by *Megasphaera* to generate SCFAs such as valerate (Huertas-Díaz et al., 2025), forming a typical metabolic cross-feeding relationship. Such cooperation may improve substrate utilization efficiency and sustain the supply of energy-yielding metabolites. Collectively, the enrichment of these functional taxa and their metabolic interactions may enhance SCFA production and utilization, thereby promoting nutrient absorption, improving immune balance, and increasing overall energy efficiency. These processes may represent an important microbial basis for the high-body-weight phenotype.

The metabolomic data further demonstrated that chickens with divergent body weights exhibited distinct metabolic regulatory patterns. The H group was primarily enriched in lipid metabolism pathways, particularly glycerophospholipid metabolism, and exhibited increased levels of metabolites such as phosphatidylcholine (PC) and phosphatidylethanolamine (PE), suggesting a greater capacity for energy storage and tissue biosynthesis (Li and Vance, 2008; van der Veen et al., 2017). In contrast, the L group exhibited a metabolic profile dominated by amino acid metabolism. Altered levels of metabolites such as citrulline, arginine, and creatine suggest increased protein turnover in these birds. However, when amino acids are utilized as energy substrates, deamination and nitrogen metabolism are required, incurring additional energetic costs and potentially diverting substrates away from protein accretion, thereby reducing overall energy efficiency (Selle et al., 2022; Macelline et al., 2025). Such a metabolic pattern is therefore less favorable for efficient biomass accumulation. The enrichment of arachidonic acid in the H group provides further evidence of enhanced lipid metabolism. As a key component of membrane phospholipids, arachidonic acid can be converted into multiple bioactive molecules involved in the regulation of energy metabolism, nutrient transport, and inflammatory responses, thereby contributing to improved nutrient utilization and intestinal homeostasis (Stables and Gilroy, 2011; Wang et al., 2021). Taken together, the differential regulation of lipid and amino acid metabolism may represent a key metabolic basis underlying body weight divergence. Broilers with higher body weight may preferentially channel excess substrates toward lipid deposition, thereby reducing nitrogen excretion and the energetic costs associated with amino acid catabolism, ultimately contributing to improved growth efficiency.

Integrative analysis of the microbiome and metabolome revealed a close interplay between the gut microbial community and host metabolic processes. The enrichment of functional taxa such as *Lactobacillus, Megasphaera*, and *Negativibacillus* in the high-body-weight group may contribute to host lipid metabolism through modulation of key metabolites, including PC, PE, and arachidonic acid. These changes may promote more efficient energy utilization and deposition, thereby supporting greater body weight. However, the precise mechanisms underlying these interactions remain to be fully elucidated. Future studies integrating fecal microbiota transplantation and multi-omics approaches will be essential to clarify the causal roles of the gut microbiota in regulating host growth and to provide a theoretical basis for optimizing livestock production performance.

## Conclusions

This study demonstrates that body weight variation in hybrid chickens is closely associated with differences in cecal microbial community structure and serum metabolomic profiles. High-body-weight chickens were characterized by the enrichment of functional taxa, including *Lactobacillus, Megasphaera*, and *Subdoligranulum*, along with elevated levels of lipid metabolites such as phosphatidylethanolamine, phosphatidylcholine, and arachidonic acid. In contrast, low-body-weight chickens exhibited higher abundances of *Colidextribacter, Pseudoflavonifractor*, and I*ntestinimonas*, along with a metabolomic profile biased toward amino acid metabolism, with significant alterations in metabolites such as arginine, citrulline, and D-proline.

In summary, this study improves our understanding of the coordinated roles of gut microbial ecology and host metabolism in shaping growth performance and provides a theoretical basis for the development of microbiome-based strategies to improve poultry production efficiency.

## Funding

This study was supported by the Key Core Technology Research Program for Mountain Agriculture in Guizhou Province, entitled “Breeding and Demonstration of Specialized Lines for ‘Laziji’ Chicken Production” (Grant No. GZNYGJHX-2025004).

## Ethics statement

All animal procedures were conducted in accordance with the Guidelines for the Care and Use of Experimental Animals issued by the Ministry of Agriculture and Rural Affairs of the People’s Republic of China. All experimental protocols were reviewed and approved by the Animal Ethics Committee of Guizhou University (Approval No. EAE-GZU-2025-E087).

## Data availability

The raw 16S rRNA gene sequencing data generated in this study have been deposited in the NCBI Sequence Read Archive (SRA) database under BioProject accession number PRJNA1464185. Other datasets supporting the findings of this study are available from the corresponding author upon reasonable request.

## CRediT authorship contribution statement

**Xudong Zhao:** Writing – review & editing, Writing – original draft, Validation, Supervision, Formal analysis. **Yingping Tian:** Writing – review & editing, Writing – original draft. **Sheng Wu:** Writing – review & editing, Data curation. **Yong Yue:** Writing – review & editing, Writing – original draft. **Yun Du:** Writing – review & editing. **Fuping Zhang:** Writing – review & editing, Writing – original draft, Supervision, Funding acquisition. **Xing Lei:** Writing – review & editing. **Zhiwen Chen:** Writing – review & editing. **Yaozhou Jiang:** Writing – review & editing. **Qinsong Liu:** Writing – review & editing.

## Disclosures

The authors declare that they have no known competing financial interests or personal relationships that could have appeared to influence the work reported in this paper.

## Appendix Supplementary materials

Image, application 1

## Footnotes

Supplementary material associated with this article can be found, in the online version, at doi:10.1016/j.psj.2026.107117.
