# Supplementary material for: Microbiomics and metabolomics reveal microbial–metabolic signatures associated with body weight variation in chickens
Source: Poult Sci. 2026 May 13;105(8):107117. doi: 10.1016/j.psj.2026.107117 (PMC13233762; doi:10.1016/j.psj.2026.107117)
Supplement: Supplementary file 1 [file mmc1.docx]

Table S1 First-round PCR reaction components

| Component | Volume (μL) | Notes |
| --- | --- | --- |
| Buffer | 10 | Reaction buffer |
| Q5 High-Fidelity DNA Polymerase | 0.2 | High-fidelity enzyme |
| High GC Enhancer | 10 | GC-rich template enhancer |
| dNTPs | 1 | Nucleotide mix |
| Forward primer (10 μM) | 1–2 | Adjustable based on template |
| Reverse primer (10 μM) | 1–2 | Adjustable based on template |
| Template DNA | 60 ng | Sample DNA |
| Total volume | 50 | -- |

Table S2 First-round PCR cycling conditions

| Step | Temperature (°C) | Time | Cycles |
| --- | --- | --- | --- |
| Initial denaturation | 95 | 5 min | 1 |
| Denaturation | 95 | 1 min | 15 |
| Annealing | 50 | 1 min | 15 |
| Extension | 72 | 1 min | 15 |
| Final extension | 72 | 7 min | 1 |

Table S3 Second-round PCR reaction components

| Component | Volume (μL) | Notes |
| --- | --- | --- |
| 2× Phusion HF Master Mix | 20 | High-fidelity PCR master mix |
| ddH₂O | 8 | Nuclease-free water |
| Forward primer (10 μM) | 1–2 | Includes adapters/barcodes |
| Reverse primer (10 μM) | 1–2 | Includes adapters/barcodes |
| First-round PCR product | 10 | Template DNA |
| Total volume | 40 | -- |

Table S4. Second-round PCR cycling conditions

| Step | Temperature (°C) | Time | Cycles |
| --- | --- | --- | --- |
| Initial denaturation | 98 | 30 s | 1 |
| Denaturation | 98 | 10 s | 10 |
| Annealing | 65 | 30 s | 10 |
| Extension | 72 | 30 s | 10 |
| Final extension | 72 | 5 min | 1 |

Table S5 Sample sequencing data processing result statistics

| Sample ID | Raw Reads | Clean Reads | Denoised Reads | Merged Reads | Non-chimeric Reads |
| --- | --- | --- | --- | --- | --- |
| FH2026 | 80143 | 73888 | 72478 | 67999 | 58447 |
| FH2027 | 79942 | 73541 | 72248 | 68242 | 58529 |
| FH2048 | 99285 | 90530 | 88667 | 84145 | 71219 |
| FH2078 | 79777 | 73822 | 72866 | 69861 | 59610 |
| FH2082 | 80245 | 74116 | 72674 | 67497 | 57087 |
| FH2187 | 80139 | 73265 | 71675 | 67747 | 58260 |
| FL2020 | 80182 | 73820 | 72464 | 67549 | 59490 |
| FL2034 | 80257 | 73393 | 71762 | 67656 | 57804 |
| FL2092 | 80140 | 73923 | 72376 | 68033 | 54490 |
| FL2111 | 79927 | 73232 | 71832 | 67152 | 54513 |
| FL2174 | 79844 | 73866 | 72422 | 68436 | 55573 |
| FL2191 | 80052 | 73647 | 72102 | 67107 | 56778 |
| MH2070 | 80025 | 73958 | 72215 | 66650 | 56818 |
| MH2106 | 79736 | 73650 | 72052 | 67354 | 56380 |
| MH2150 | 80099 | 74074 | 72276 | 66947 | 56950 |
| MH2156 | 79933 | 73988 | 72159 | 65613 | 56039 |
| MH2158 | 80096 | 73975 | 72585 | 69042 | 58174 |
| MH2165 | 80098 | 74150 | 72467 | 66335 | 56819 |
| ML2005 | 80086 | 73794 | 72101 | 66743 | 58613 |
| ML2064 | 80094 | 73300 | 71938 | 67292 | 57196 |
| ML2073 | 80189 | 74062 | 72309 | 66947 | 56824 |
| ML2130 | 79805 | 73612 | 72191 | 68573 | 59951 |
| ML2167 | 80368 | 73820 | 72377 | 67878 | 54653 |
| ML2178 | 80272 | 73821 | 72306 | 67733 | 58917 |

Sample ID indicates the sample name. Raw Reads represent the number of original sequencing reads obtained from sequencing. Clean Reads indicate the number of high-quality reads after quality control of the raw sequences. Denoised Reads refer to the number of reads obtained after denoising the clean reads. Merged Reads represent the number of sequences generated after merging the denoised reads based on overlap regions. Non-chimeric Reads indicate the final number of sequences remaining after the removal of chimeric sequences.

Table S6 Identification of metabolites that differed significantly in the serum of different body weights of hybrid chicken（VIP > 1.0，FC > 1.0，*P-value* < 0.05）.

| Name | log2FC | *P-*value | VIP | regulated |
| --- | --- | --- | --- | --- |
| Citrulline | -1.804004 | 0.007748 | 2.624935 | down |
| Argininic acid | -1.440639 | 0.015492 | 2.31459 | down |
| Creatine | -1.213789 | 0.016229 | 2.198963 | down |
| Butyrylcarnitine | -1.153066 | 0.038963 | 1.705537 | down |
| L-Acetylcarnitine | -1.025719 | 0.008675 | 1.906027 | down |
| 3-Dehydroxycarnitine | -0.984239 | 0.036401 | 1.942001 | down |
| Pseudouridine | -0.957744 | 0.043165 | 1.692017 | down |
| 2-O-(5,8,11,14,17-Eicosapentaenoyl)-1-O-hexadecylglycero-3-phosphocholine | -0.873923 | 0.044723 | 1.724048 | down |
| PC (P-18:0/14:0) | -0.853537 | 0.015267 | 1.858674 | down |
| Acetylglycine | -0.853483 | 0.025722 | 1.784571 | down |
| 2-Hydroxybutyric acid | -0.847614 | 0.029864 | 1.584164 | down |
| N-Acetylserine | -0.835071 | 0.031487 | 1.418711 | down |
| 1-Methylguanine | -0.822231 | 0.043189 | 1.673548 | down |
| 2-Hydroxy-3-methylbutyric acid | -0.807396 | 0.034201 | 1.778535 | down |
| PC (P-18:0/16:0) | -0.805386 | 0.031383 | 1.69012 | down |
| L-Allothreonine | -0.704848 | 0.011415 | 2.027266 | down |
| Oxidized-adrenal-ferredoxin | -0.614387 | 0.030126 | 1.805808 | down |
| 5,6-Dihydrouridine | -0.609105 | 0.021283 | 1.725379 | down |
| Pentanenitrile | -0.598763 | 0.026123 | 1.319242 | down |
| Glycerophosphocholine | -0.575316 | 0.04886 | 1.302832 | down |
| Mafenide (Acetate) | -0.548597 | 0.029949 | 1.546223 | down |
| L-Carnitine | -0.522333 | 0.037089 | 1.692539 | down |
| Beta-Alanine | -0.460845 | 0.03322 | 1.732907 | down |
| Propionic acid | -0.457396 | 0.013041 | 2.175789 | down |
| Aminoacetone | -0.441582 | 0.02896 | 1.688909 | down |
| Pyrrolidine | -0.436413 | 0.016988 | 1.878913 | down |
| (+/-)-1-Acetoxy-1-ethoxyethane | -0.411515 | 0.04695 | 1.713819 | down |
| L-allo-isoleucine | -0.317747 | 0.008832 | 2.030269 | down |
| D-Proline | -0.310992 | 0.012929 | 2.224747 | down |
| L-Tryptophan | -0.278293 | 0.036559 | 1.862603 | down |
| Raddeanin A | -0.275042 | 0.023618 | 2.106229 | down |
| L-Valine | -0.242188 | 0.015679 | 1.947174 | down |
| Glycolic acid | -0.187239 | 0.009397 | 1.984401 | down |
| D-Phenylalanine | -0.161699 | 0.032248 | 1.957602 | down |
| phenethanolamine | -0.152653 | 0.035915 | 1.946106 | down |
| Pelargonic acid | 0.191966 | 0.016929 | 1.899163 | up |
| PC (22:4(7Z,10Z,13Z,16Z)/14:0) | 0.259481 | 0.011764 | 1.682188 | up |
| PC (22:2(13Z,16Z)/18:2(9Z,12Z)) | 0.262369 | 0.043033 | 1.643564 | up |
| PC (20:4(8Z,11Z,14Z,17Z)/20:3(5Z,8Z,11Z)) | 0.282119 | 0.049394 | 1.323102 | up |
| FAPy-adenine | 0.291382 | 0.023728 | 1.713879 | up |
| PC (18:1(9Z)/18:3(6Z,9Z,12Z)) | 0.301176 | 0.007135 | 1.921348 | up |
| PC (20:2(11Z,14Z)/14:0) | 0.305222 | 0.037187 | 1.845362 | up |
| N-Acetylgalactosamine 6-sulfate | 0.352265 | 0.02275 | 1.844391 | up |
| PC (22:5(4Z,7Z,10Z,13Z,16Z)/14:0) | 0.354445 | 0.029551 | 1.735147 | up |
| Methoxypyrazine | 0.38578 | 0.019817 | 2.017809 | up |
| PC (22:5(4Z,7Z,10Z,13Z,16Z)/18:0) | 0.408887 | 0.045121 | 1.939036 | up |
| PC (22:6(4Z,7Z,10Z,13Z,16Z,19Z)/20:2(11Z,14Z)) | 0.432188 | 0.014323 | 1.81695 | up |
| 5-Methylcytosine | 0.448552 | 0.024939 | 1.710239 | up |
| PC (20:3(8Z,11Z,14Z)/20:1(11Z)) | 0.455515 | 0.007811 | 2.265882 | up |
| PC (22:4(7Z,10Z,13Z,16Z)/16:0) | 0.467685 | 0.027363 | 1.897851 | up |
| N-Nitroso-pyrrolidine | 0.47988 | 0.002982 | 2.190224 | up |
| PC (20:1(11Z)/14:1(9Z)) | 0.612883 | 0.020644 | 2.059605 | up |
| PC (16:0/14:0) | 0.626801 | 0.034551 | 1.746282 | up |
| Arachidonic Acid (peroxide free) | 0.641232 | 0.004319 | 2.018447 | up |
| Galanthamine N-Oxide | 0.642184 | 0.003339 | 2.164171 | up |
| PC (18:2(9Z,12Z)/20:5(5Z,8Z,11Z,14Z,17Z)) | 0.771723 | 0.005516 | 2.227991 | up |
| LY294002 | 1.049002 | 0.001308 | 2.213803 | up |
| PE (22:4(7Z,10Z,13Z,16Z)/16:1(9Z)) | 1.161923 | 0.001743 | 2.484044 | up |
| Gallic acid | 1.204737 | 0.020167 | 2.318714 | up |
| 2,6-Dihydroxybenzoic acid | 1.867244 | 0.011466 | 2.358489 | up |

Note: Column 1 is the English name of the metabolite, followed by the value of the log2 of the multiplicity of differences, the *P*-value of the *t*-test, the VIP value of the OPLS-DA model, and the up- and down-regulation information.


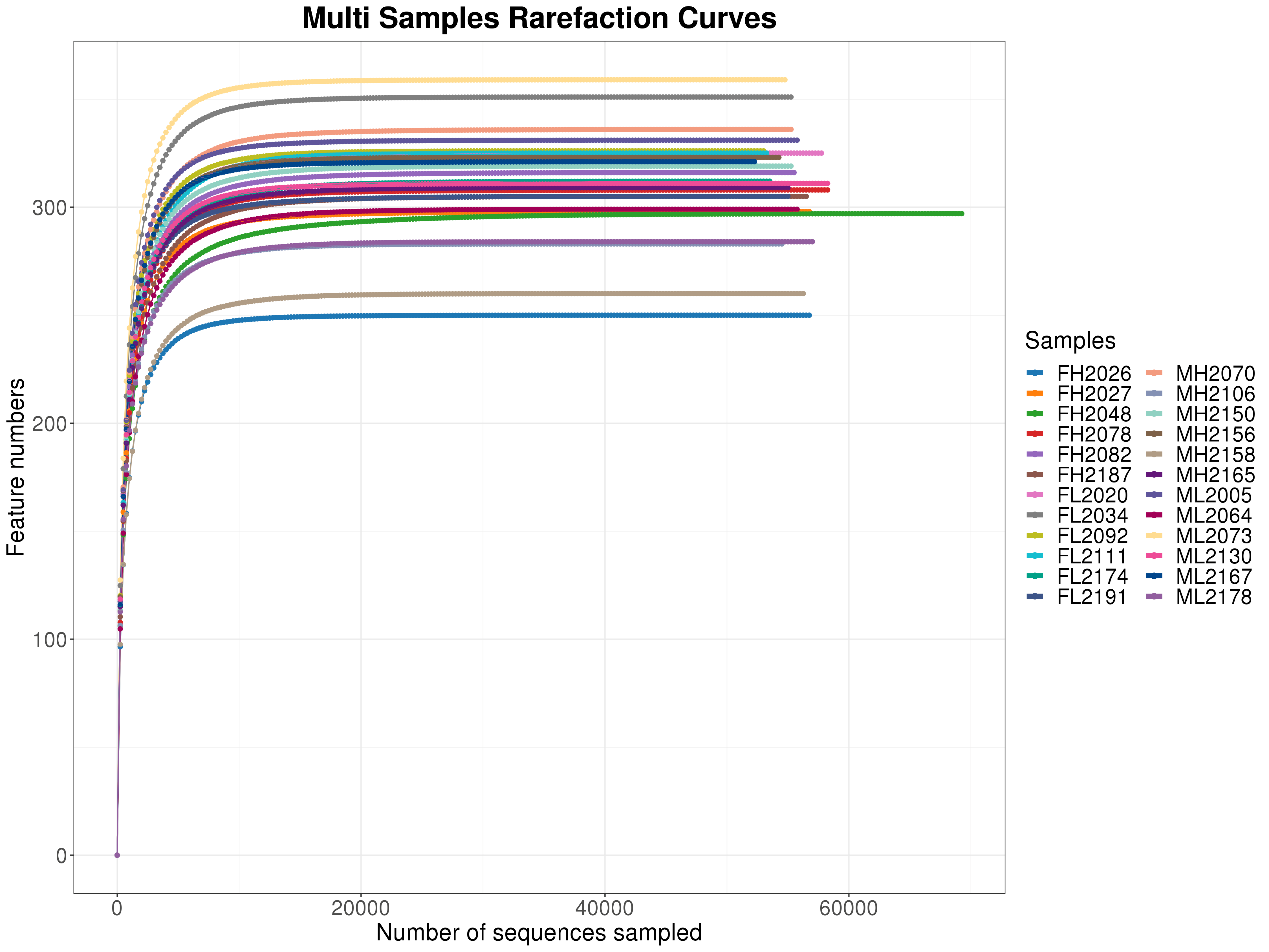


Figure S1 Multi-Sample rarefaction curves

Note: The coordinates are the number of randomly selected sequencing strips. The vertical coordinate is the number of features obtained based on the number of sequencing strips. Each curve represents a sample marked with a different colour.
